# Clinical features in primary care electronic records before diagnosis of ankylosing spondylitis: a nested case-control study

**DOI:** 10.1186/s12875-020-01149-2

**Published:** 2020-05-06

**Authors:** Mohammed T. Bashir, Lisa Iversen, Christopher Burton

**Affiliations:** 1 Medical School, University of Aberdeen, Aberdeen, UK; 2 Institute of Applied Health Sciences, University of Aberdeen, Aberdeen, UK; 3 Academic Unit of Primary Medical Care, University of Sheffield, Samuel Fox House, Northern General Hospital, Sheffield, S5 7AU UK

**Keywords:** Ankylosing spondylitis, Diagnosis, Primary care, Electronic health records

## Abstract

**Background:**

Ankylosing spondylitis (AS) often has a long period from first symptom presentation to diagnosis. We examined the occurrence of symptoms, prescriptions and diagnostic tests in primary care electronic records over time prior to a diagnosis of AS.

**Methods:**

Nested case-control study using anonymised primary care electronic health records from Scotland. Cases were 74 adults with a first diagnosis of AS between 2000 and 2010. Controls were matched for age, sex and GP practice: (a) 296 randomly selected adults (b) 169 adults whose records contained codes indicating spinal conditions or symptoms.

We extracted clinical features (symptoms, AS-related disorders, prescriptions and diagnostic tests). Conditional logistic regression was used to examine the association between clinical features (both individually and in combinations) and diagnosis of AS. We examined the associations between clinical features and diagnosis over time prior to diagnosis.

**Results:**

Several new composite pointers were predictive of AS: including distinct episodes of axial pain separated by more than 6 months (OR 12.7, 95% CI 4.7 to 34.6); the occurrence of axial pain with and tendon symptoms within the same year (OR 21.7, 95% CI 2.6 to 181.5); and the co-occurrence (within 30 days) of axial pain and a prescription for nonsteroidal anti-inflammatory drug (OR 10.4, 95%CI 4.9 to 22.1). Coded episodes of axial pain increased steadily over the 3 years before diagnosis. In contrast, large joint symptoms and enthesopathy showed little or no time trend prior to diagnosis.

**Conclusions:**

We identified novel composite pointers to a diagnosis of AS in GP records. These may represent valuable targets for diagnostic support systems.

## Background

Ankylosing Spondylitis (AS) is an uncommon rheumatological condition in primary care, for which there is often a long time between consultation and diagnosis [1, 2]. As with other conditions in which a long period between first consultation and diagnosis is often seen, symptoms of AS (such as spinal pain, stiffness and fatigue) are both non-specific and frequently occuring [2, 3]. This often leads to primary care doctors assigning the symptoms of AS to more common back pain conditions.

Most research on the clinical features of AS in primary care has focused on the characteristics of back pain. Inflammatory features such as worsening in the second half of the night, causing stiffness on waking and relief by exercise [4–8] have high sensitivity but relatively low specificity for AS. We considered the possibility of using data in electronic records to support earlier diagnosis of AS. While records may include codes for back pain, they do not currently include searchable information about the characteristics of pain. However other features in records may act as proxy markers for these pain charateristics. For instance the association in time between back pain and prescription of non-steroidal anti-inflammatory drugs (NSAID) may suggest inflammatory back pain (which often responds well to NSAIDs). While such knowledge-derived features [9] are not immediately present in electronic records, they can be constructed [10, 11]. One very recent study has used machine learning based on single items in a clinical care database but still found only low predictive values [12]..

We aimed to (a) construct enriched datasets from electronic health records which contained conventional and composite features potentially predictive of AS; (b) examine the association of these features with a subsequent diagnosis of AS in a nested case-control study; (c) examine the relationship of these features to diagnosis at different time periods before the date of diagnosis.

## Methods

### Data source

We analysed data from the Practice Team Information (PTI) database, a subset of the Primary Care Clinical Informatics Unit Research database held by the University of Aberdeen. The PTI database is comprised of pseudonymised electronic health records which were collected between 1996 and 2010 from approximately 224,000 patients registered with a primary care physician in Scotland. It is broadly representative of the Scottish population with regards to age, sex, deprivation and geographical location in terms of the ratio of urban: rural practices [13]. Practices which contributed their data to the PTI project were expected to record every clinical encounter using Read codes for clinical diagnoses and / or, main reasons for consultation. Diagnostic codes entered during and before the study dates were included in the data. All GP prescriptions were automatically recorded throughout the database period. Procedures for the routine recording and coding of diagnostic investigations changed over the database period as electronic linkages between laboratories and GP practices developed. Investigations were present in the data more in later years of the database period. The study was approved by the Primary Care Clinical Informatics Unit (PCCIU) team in keeping with PCCIU and local ethical committee procedures.

### Populations

We conducted a nested case-control study. Cases were patients whose first recorded diagnosis of AS was between 1/1/2000 and 31/12/2010 and who were aged between 18 and 50 years at the time of diagnosis. We excluded patients whose first recorded diagnosis occurred within 1 year of registering with their GP practice as (a) it was possible that this represented the coding of an earlier diagnosis for the purposes of updating record summaries i.e. a prevalent rather than incident case of AS (b) it did not allow sufficient period of time in which relevant data before diagnosis could be examined for features predictive of AS. We then excluded patients who had been prescribed a disease modifying anti-rheumatic drug (sulphasalazine or methotrexate) more than 1month prior to their coded diagnosis. We did this in order to ensure that our analysis was limited to incident cases rather than prevalent cases in whom an earlier diagnosis had not been coded at the time it was made.

Population controls who did not have a diagnosis of AS at the index date were identified electronically from the database for each case. Controls were individually matched on age, sex and GP practice. Where more than four matched controls were available for a given case the computer randomly selected four. A second control group comprised patients with codes for other spinal diagnoses including degenerative, mechanical and intervertebral disc disorders or for a symptom of axial pain, but with no recorded diagnosis of AS. These controls were also electronically selected for each case and individually matched by age (within 2 years), sex and GP practice, with up to four symptomatic controls per case. We defined the index date for cases as the date of diagnosis of AS and for controls as the date of diagnosis of AS in the matched case.

### Data extraction and preparation

For all cases and controls, data were extracted which detailed the dates of consultation for particular symptoms, disorders, tests and procedures and drugs prescribed. Table 1 lists the key data extracted and the categories into which we grouped related items.

**Table 1:** Types of data extracted and categories which individual codes were mapped to

Data type	Data type categories
Spinal symptoms and disorders	Axial pain Other spinal diagnoses e.g.cervical spondylosis, mechanical back pain Sciatica^a^
Musculoskeletal and AS- related features	Enthesopathy and other tendon disorders Large joint symptoms / disorders e.g. knee pain Other joint symptoms / disorders e.g. foot pain Iritis Urethral symptoms Fatigue
Other related disorders	Inflammatory Bowel Disease Inflammatory Arthritis (including psoriatic arthropathy)
Diagnostic tests and procedures	Full blood count Erythrocyte Sedimentation Rate (ESR) Thyroid Function^b^ X-ray (spine / pelvis) Computerised Tomography Magnetic Resonance Imaging
Treatments	Non-steroidal anti-inflammatory drugs (NSAIDs) Analgesics (up to codeine) Opioids (tramadol and more potent) Tricyclic antidepressants^c^ Selective Serotonin Re-uptake Inhibitors (SSRI)^d^

^a^Sciatica included to look for back pain in absence of sciatica

^b^Thyroid function included as used in non-specific / fatigue work up

^c^Tricylics are typically prescribed for chronic pain rather than for depression

^d^SSRIs are typically prescribed for anxiety or depression rather than for chronic pain

As well as examining various individual features e.g. axial pain, we enriched the data by calculating when a number of composite features had occurred e.g. axial pain occurring within 30 days of a prescription for a non-steroidal anti-inflammatory drug (NSAID). We specified composite features according to one of three relationships: proximity (where two features occurred within a given number of days of each other), separate (where two consecutive instances of the same features occurred more than a given number of days apart), and exclusive (where one code occurred and another was not present). We used the separate composite features in order to identify discrete episodes as opposed to a single episode comprising multiple instances of a feature.

For each feature (single and composite) we ascertained its presence in the record of each individual at any time in the record, and during a series of overlapping three-year time windows set at different intervals from the index date (for diagnosis or matching). We defined the windows using intervals between the end of the window and the index date of 0, 3, 6, 12, 18, 24 and 36 months. We then examined the appearance of statistical associations between available information in the record and diagnosis over time by comparing the same measure in different windows. The purpose of this was to differentiate between features which were present long before diagnosis (and might thus indicate missed diagnostic opportunities) and those which appeared only shortly before diagnosis (and may thus have triggered referral).

### Analysis of association of features and patterns with diagnosis

We carried out conditional logistic regression to examine the association between each feature (conventional or composite) and the diagnosis of AS. Each feature was reported as either present or absent within the time period. Rather than use counts of how often a feature occurred, we used the “separated” composite variables to indicate multiple episodes. Analyses were reported as the odds ratio, OR (with 95% confidence intervals, CI). All analyses were conducted in R 3.6 [14].

We conducted the analysis separately with population and symptomatic control groups. For the time window analysis, we limited the data to patients who had been registered with their practice for at least 1year before the beginning of the relevant gap prior to diagnosis. We plotted the odds ratios for each feature at each of the six different time gaps in order to visualise the appearance of predictive features over time.

## Results

### Patient characteristics

There were 74 newly diagnosed cases who met the study criteria. The annual number of diagnoses was broadly similar between 2000 and 2006 (representing an incidence of approximately 4/100,000 registered patients per year) but fell after 2006 – this coincided with a progressive reduction in the size of the database as GP practice computer systems were replaced.

Cases were matched to 296 population controls and 169 symptomatic controls. 53 cases (72%) were men and median age at diagnosis of AS was 37 years (interquartile range 31 to 43).

### Data quality

54 cases (73%) were registered with the same GP practice (and therefore had continuous records in the PTI database) for at least 6 years before diagnosis. Similar proportions were seen for population and symptomatic controls (81 and 72% respectively). A code for one or more prescription of an appropriate treatment (e.g. a NSAID) was present for 68 cases (92%). Diagnostic tests were coded less often – any relevant diagnostic code (such as for a full blood count, ESR or x-ray) was present in only 30 cases (41%).

### Occurrence of diagnostic features

The numbers and proportions of patients with at least one instance of each feature, either in the 3 years prior to the index date or at any time are shown in Table 2 (vs. population controls) and Table 3 (vs. symptomatic controls). Tables 2 & 3 also show the ORs (with 95% CIs) for the two comparisons.

**Table 2:** Numbers, proportions and odds ratios (95% CI) for features in cases of ankylosing spondylitis compared with population controls

	Occurrence of features in 3 years before index date						Occurrence of features at any time before index date					
	Cases (*N* = 74)		Controls (*N* = 296)				Cases (*N* = 74)		Controls (*N* = 296)			
**Specific features**	N	*%*	N	*%*	OR	(95% CI)	N	*%*	N	*%*	OR	(95% CI)
Axial pain	39	52.7	31	10.5	9.8	(5.1, 18.9)	49	66.2	70	23.7	7.5	(4.0, 14.2)
Other spine diagnosis	16	21.6	17	5.7	5.1	(2.3, 11.3)	22	29.7	33	11.2	3.7	(1.9, 7.3)
Sciatica	5	6.8	8	2.7	2.8	(0.8, 9.3)	9	12.2	18	6.1	2.3	(0.9, 5.8)
Large joint symptom	14	18.9	25	8.5	2.7	(1.3, 5.8)	22	29.7	47	15.9	2.4	(1.3, 4.4)
Other joint symptom	5	6.8	4	1.4	6.0	(1.4, 25.5)	7	9.5	10	3.4	3.4	(1.2, 9.8)
Tendon disorders	9	12.2	12	4.1	3.4	(1.3, 8.7)	16	21.6	21	7.1	3.6	(1.7, 7.3)
Iritis	8	10.8	1	0.3	32.0	(4.0, 255.9)	12	16.2	3	1.0	16.0	(4.5, 56.7)
Urethral symptoms	2	2.7	1	0.3	–	–	3	4.1	8	2.7	1.5	(0.4, 6.0)
Fatigue	10	13.5	24	8.1	1.9	(0.8, 4.2)	12	16.2	39	13.2	1.3	(0.6, 2.8)
Inflamm. Bowel disease	7	9.5	4	1.4	7.0	(2.1, 23.9)	12	16.2	13	4.4	4.9	(2.0, 12.3)
Other Inflamm. arthritis	3	4.1	2	0.7	–	–	5	6.8	4	1.4	–	–
X-ray spine	6	8.1	0	0.0	–	–	7	9.5	2	0.7	–	–
X-ray pelvis	2	2.7	0	0.0	–	–	2	2.7	0	0.0	–	–
Full Blood Count	12	16.2	19	6.4	5.5	(1.8, 16.4)	15	20.3	23	7.8	9.2	(2.5, 33.4)
ESR	6	8.1	5	1.7	14.9	(1.7, 127.8)	7	9.5	6	2.0	9.7	(2.0, 47.9)
MRI scan	0	0.0	2	0.7	–	–	0	0.0	3	1.0	–	–
NSAIDs	52	70.3	60	20.3	12.9	(6.3, 26.8)	57	77.0	93	31.4	13.3	(5.9, 29.9)
Analgesics	37	50.0	43	14.5	6.0	(3.3, 10.8)	41	55.4	73	24.7	4.4	(2.4, 7.8)
Opioids	8	10.8	5	1.7	7.5	(2.2, 25.0)	10	13.5	8	2.7	7.9	(2.4, 25.7)
Tricyclic antidepressants	10	13.5	15	5.1	2.9	(1.3, 6.8)	12	16.2	18	6.1	3.2	(1.4, 7.3)
SSRI antidepressants	5	6.76	33	11.2	0.56	(0.2, 1.5)	12	16.22	13	4.39	4.91	(2.0, 12.3)

Features: *Large Joint symptom* codes relating to large limb joints (shoulder-wrist, hip-ankle); *Other joint symptoms* codes relating to joints distal to wrist/ ankle; *ESR* Erythrocyte sedimentation rate, *MRI* Magentic resonance imaging, *NSAID* Non-steroidal anti-inflammatory drugsm, *Analgesics* paracetamol, codeine; Opioids opioid drugs of the potency of tramadol or higher, *SSRI* Selective Serotonin Reuptake Inhibitor & related antidepressants**;** Index date: date of diagnosis for cases, date of diagnosis of matched case for controls

**Table 3:** Numbers, proportions and odds ratios (95% CI) for features in cases of ankylosing spondylitis compared with symptomatic controls

	Occurrence of features in 3 years before index date						Occurrence of features at any time before index date					
	Cases (*N* = 74)		Controls (*N* = 169)				Cases (*N* = 74)		Controls (*N* = 169)			
**Specific features**	N	*%*	N	*%*	OR	(95% CI)	N	*%*	N	*%*	OR	(95% CI)
Axial pain	39	52.7	93	55.0	1.0	(0.6, 1.8)	49	66.2	146	86.4	0.3	(0.2, 0.6)
Other spine diagnosis	16	21.6	25	14.8	1.8	(0.8, 3.9)	22	29.7	40	23.7	1.4	(0.7, 2.7)
Sciatica	5	6.8	19	11.2	0.6	(0.2, 1.7)	9	12.2	31	18.3	0.7	(0.3, 1.4)
Large joint symptom	14	18.9	14	8.3	2.1	(0.9, 4.8)	22	29.7	34	20.1	1.5	(0.8, 2.9)
Other joint symptom	5	6.8	4	2.4	2.7	(0.6, 12.5)	7	9.5	6	3.6	2.8	(0.8, 10.3)
Tendon disorders	9	12.2	10	5.9	2.1	(0.8, 5.5)	16	21.6	21	12.4	1.7	(0.8, 3.6)
Iritis	8	10.8	0	0.0	–	–	12	16.2	0	0.0	–	–
Urethral symptoms	2	2.7	1	0.6	–	–	3	4.1	4	2.4	1.5	(0.3, 6.8)
Fatigue	10	13.5	14	8.3	2.1	(0.8, 5.7)	12	16.2	22	13.0	1.3	(0.5, 3.1)
Inflamm. Bowel disease	7	9.5	2	1.2	12.0	(1.5, 99.4)	12	16.2	9	5.3	3.2	(1.3, 7.8)
Other Inflamm. arthritis	3	4.1	2	1.2	–	–	5	6.8	4	2.4	2.5	(0.7, 9.5)
X-ray spine	6	8.1	2	1.2	12.6	(1.5, 106.7)	7	9.5	3	1.8	13.7	(1.6, 113.6)
X-ray pelvis	2	2.7	0	0.0	–	–	2	2.7	1	0.6	–	–
–	–	16.2	18	10.7	3.2	(1.0, 10.8)	15	20.3	23	13.6	4.6	(1.2, 17.5)
ESR	6	8.1	1	0.6	14.3	(1.7, 120.8)	7	9.5	2	1.2	14.5	(1.8, 120.7)
MRI scan	NA	NA	NA	NA	–	–	0	0.0	2	1.2	–	–
NSAIDs	52	70.3	70	41.4	3.6	(1.8, 7.1)	57	77.0	94	55.6	3.1	(1.4, 6.6)
Analgesics	37	50.0	53	31.4	2.0	(1.1, 3.6)	41	55.4	77	45.6	1.4	(0.8, 2.5)
Opioids	8	10.8	10	5.9	1.8	(0.7, 4.7)	10	13.5	13	7.7	1.8	(0.8, 4.2)
Tricyclic antidepressants	10	13.5	14	8.3	1.6	(0.7, 3.8)	12	16.2	20	11.8	1.3	(0.6, 3.0)
SSRI antidepressants	5	6.76	24	14.2	0.37	(0.1, 1.1)	9	12.16	33	19.5	0.51	(0.2, 1.2)

Features: *Large Joint symptom* codes relating to large limb joints (shoulder-wrist, hip-ankle); *Other joint symptoms* codes relating to joints distal to wrist/ ankle; *ESR* Erythrocyte sedimentation rate, *MRI* Magentic resonance imaging, *NSAID* Non-steroidal anti-inflammatory drugs, *Analgesics* paracetamol, codeine, *Opioids* opioid drugs of the potency of tramadol or higher, *SSRI* Selective Serotonin Reuptake Inhibitor & related antidepressants**;** Index date: date of diagnosis for cases, date of diagnosis of matched case for controls

As expected axial pain was more common in cases than population controls (OR 9.8, 95% CI 5.1 to 18.9) but not than symptomatic controls (OR 1.0, 95% CI 0.6 to 1.8) in the 3 years period before the index date. Tendon related disorders and iritis were both more common in cases than population controls (OR 3.4, 95% CI 1.3 to 8.7 and OR 32.0, 95% CI 4.0 to 255.9) but were recorded in only 21 and 16% of cases respectively. Urethral symptoms were infrequently recorded in all groups. Fatigue was not more common in cases when compared to population and symptomatic controls (OR 1.9, 95% CI 0.8 to 4.2 and 2.1, 95% CI 0.8 to 5.7) respectively. A history of inflammatory bowel disease was present in 16% of cases at any time before diagnosis. Codes indicating recording of x-rays and MRI scans were rare among cases and controls.

### Occurrence of prescribed treatments

In both the population and the symptomatic group comparisons, both analgesics (OR 6.0, 95% CI 3.3 to 10.8 and OR 2.0, 95% CI 1.1 to 3.6) and NSAIDS (OR 12.9, 95% CI 6.3 to 26.8 and OR 3.6, 95% CI 1.8 to 7.1) were more commonly prescribed in the 3 year period before the index date to cases than controls. Prescriptions of tricyclic antidepressants, typically prescribed for chronic pain, were more common compared to population controls, unlike prescriptions for other antidepressants.

### Composite features

Table 4 shows the number and proportion of patients with at least one instance of each of the composite features over the 3 years before date of diagnosis/ matching. Several composite features appeared relatively infrequently. Only three occurred in more than 15% of cases: distinct episodes of axial pain separated by more than 6 months (OR 12.7, 95% CI 4.5 to 34.6); the occurrence of axial pain with and tendon symptoms within the same year (OR 21.7, 95% CI 2.6 to 181.5); and the co-occurrence (within 30 days) of axial pain and a prescription for nonsteroidal anti-inflammatory drug (OR 10.4, 95% CI 4.9 to 22.1).

**Table 4:** Numbers, proportions and odds ratios (95% CI) for composite features in the three years before diagnosis of AS/index date

			Comparison with population controls				Comparison with symptomatic controls			
	Cases (*N* = 74)		Controls (*N* = 296)				Controls (*N* = 169)			
Composite features	N	*%*	N	*%*	OR	(95% CI)	N	*%*	OR	(95% CI)
Axial pain within 30 days of NSAID	29	39.2	20	6.8	10.4	(4.9, 22.1)	45	26.6	1.8	(0.9, 3.6)
Axial pain within 360 days of large joint symptom	9	12.2	8	2.7	7.2	(2.2, 24.0)	11	6.5	1.5	(0.5, 4.2)
Axial pain separated by 180 days	17	23.0	7	2.4	12.7	(4.7, 34.6)	17	10.1	2.8	(1.3, 5.9)
Axial pain separated by 360 days	10	13.5	5	1.7	8.0	(2.7, 23.4)	11	6.5	2.2	(0.8, 5.6)
Axial pain, without ever sciatica	36	48.7	27	9.1	8.9	(4.7, 16.9)	82	48.5	1.1	(0.6, 1.9)
Tendon disorders separated by 180 days	1	1.4	1	0.3	–	–	0	0.0	–	–
Tendon disorder within 360 days of axial pain	6	8.1	2	0.7	21.7	(2.6, 181.5)	5	3.0	2.7	(0.8, 9.0)
FBC within 90 days of axial pain	7	9.5	4	1.4	8.6	(2.2, 33.6)	10	5.9	2.2	(0.7, 7.3)
ESR within 180 days of axial pain	4	5.4	0	0.0	–	–	1	0.6	–	–
Iritis within 360 days of axial pain	5	6.8	0	0.0	–	–	0	0.0	–	–

Feature names follow the format X *relationship* Y where relationship is defined as follows X within N days of Y – any occurrence of X within N days of any occurrence of Y (in either order)

X separated by > N days – two consecutive occurrences of X separated by more than N days

X without ever Y – one or more occurrences of X and no occurrences of Y

### Occurrence of diagnostic features over the time prior to diagnosis

**Fig. 1:**
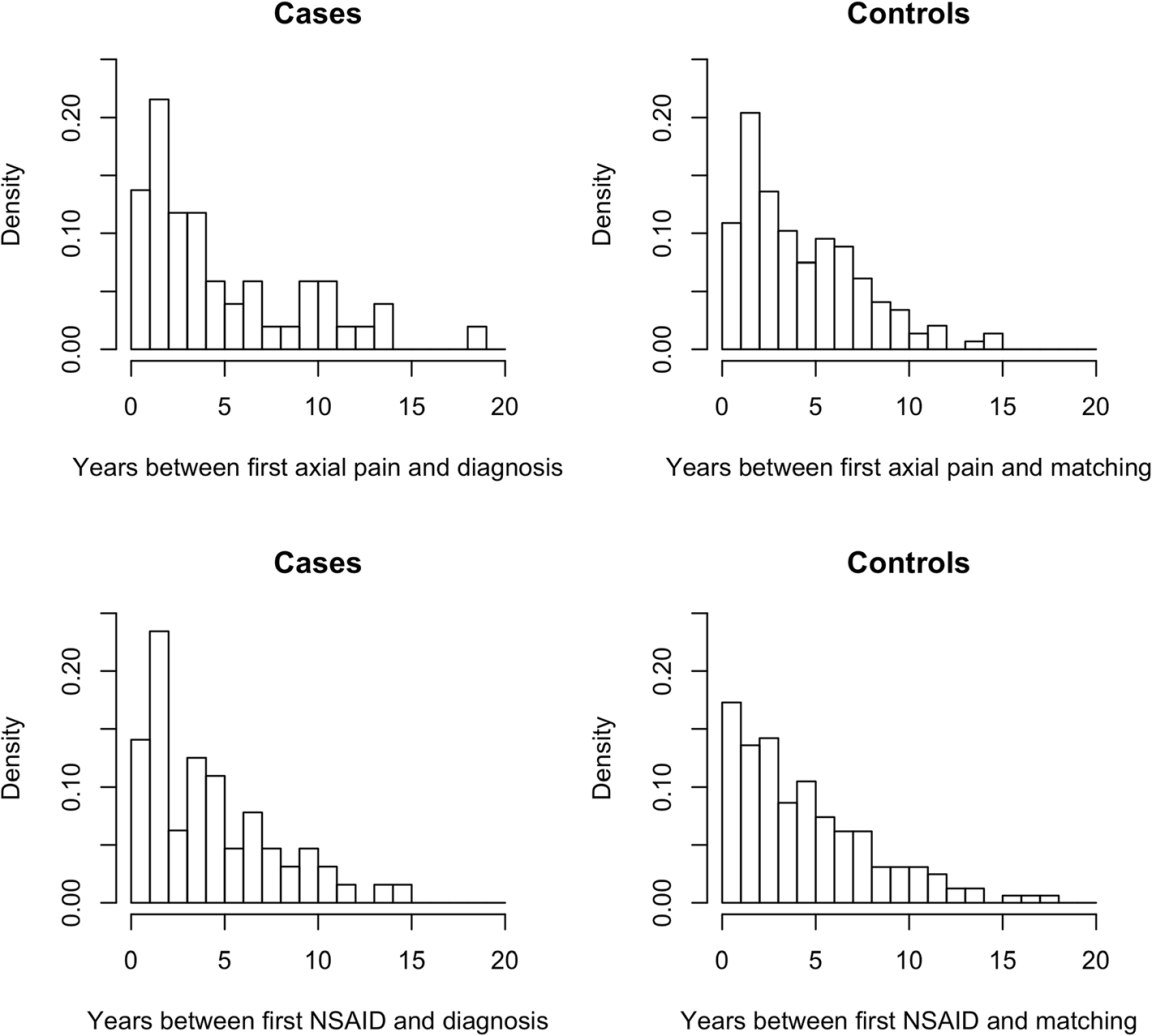
Histograms of number of years before diagnosis when back pain and NSAID prescription first present in electronic records

**Fig. 2:**
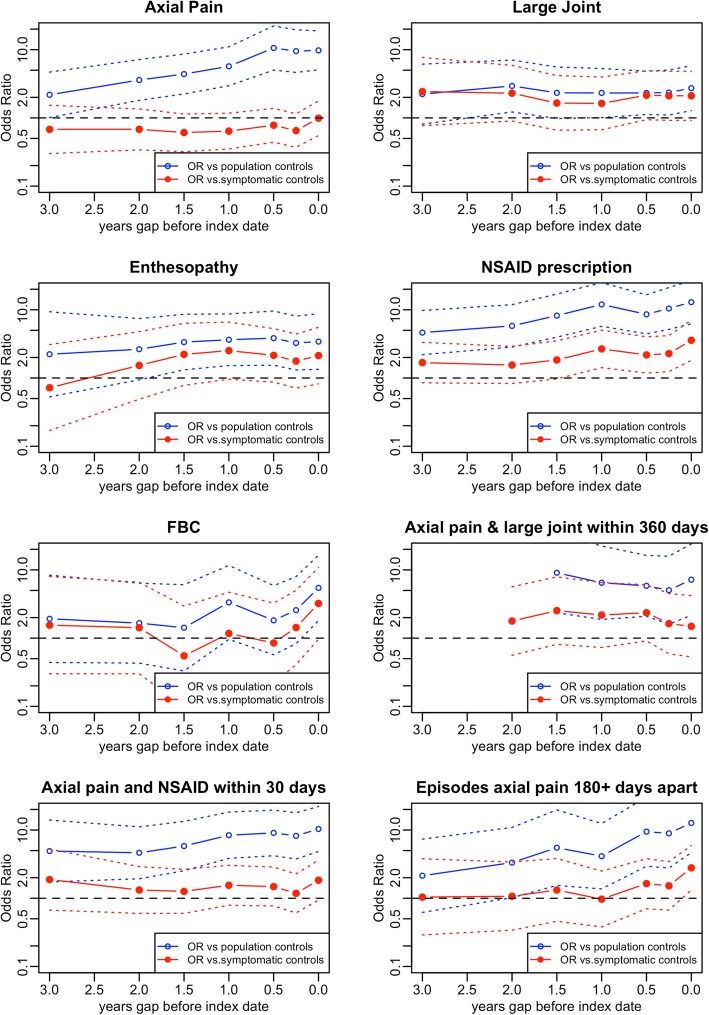
Plots of odds ratio for individual features over three years, by gap between the end of the three year window and the date of diagnosis / matching. Dotted lines indicate 95% confidence intervals for odds ratios

Figure 1 shows histograms of the number of years between first episode of back pain or NSAID prescription and diagnosis (or matching) for cases and symptomatic controls. The median time between first coded episode of back pain and diagnosis of AS was 4 years (interquartile range 2 to 7). For the same patients the median time between first prescription for a NSAID was 4 years (interquartile range 2 to 6). Fig. 2 shows plots of eight diagnostic features, showing the ORs for three-year time windows with different intervals between the end of the three-year window and the diagnosis / matching date. Each plot compares cases with matched population controls (in blue) and matched symptomatic controls (in red). In all plots, 95% confidence intervals are indicated by dotted lines. The comparison with population controls demonstrates the development of features over time. The comparison with symptomatic controls indicates whether features have different predictive value in diagnosing symptomatic patients at different stages.

The plot for axial pain shows that the odds ratio for coded episodes of axial pain rose steadily from the 3 year period ending 3 years before diagnosis to the 3 year period ending at the time of diagnosis when compared to population controls. On the other hand, the plots for large joint symptoms and (in the 2 years prior to diagnosis) enthesopathy suggests little or no time trend. The combination of axial pain and large joint symptoms – while relatively infrequent – shows a strong signal beginning at least 2 years before diagnosis.

## Discussion

### Summary of main findings

This study demonstrates new and potentially useful composite features within electronic health records, such as two or more distinct episodes of axial pain, which appear to have predictive value and may in turn lead to earlier diagnosis.

### Strengths and limitations

Our choice of features as pointers used principles of selection based on expert input [15] and methods of data consolidation and aggregation which have been developed for use with clinical data sources other than GP records [10, 16]. This sequence of steps is broadly comparable with other recent approaches to the summarisation of clinical data [16, 17]. We used an established anonymised GP record set which contained both diagnostic and symptom codes using the Read code format as well as prescribing data which means that the method is transferrable to other datasets of primary care data and potentially into clinical use.

There were limitations relating to the data. The first was the small number of incident cases of AS. This meant that confidence intervals were wide and it is possible that we lacked statistical power to detect some potentially meaningful associations. The data was from stand-alone primary care records with no linkage to secondary care records so we could not assess the reliability of GPs’ diagnosis of AS, however in our experience GP practices tend not to code such diagnoses without specialist opinion and in a recent US study a over 80% of a sample of coded diagnoses of AS were confirmed on chart review [18]. It should be noted that the annual incidence (approximately 4 per 100,000 per year in adults aged 18–60 years) is compatible with the lifetime prevalence of approximately 15 per 10,000 observed in other studies [19, 20]. The diagnostic criteria for AS and the wider spondyloarthropathies evolved during the exposure time period [21] and it is increasingly recognised that disorders in the spondyloarthritis spectrum are much more common than full AS [22].

The data on symptoms and investigations were more sparse than anticipated with only around half of cases having back pain coded in the 3 years prior to diagnosis. This probably reflects the limited use of symptom codes by GPs, even in this database where a reason for consultation was meant to be given for each attendance. For those cases where a code for axial pain was entered, there were not long periods of GPs issuing NSAID prescriptions prior to a symptom code. The use of diagnostic tests was under-reported in the database, particularly until around 2006 when the direct importing of laboratory tests into electronic records began being used widely in contributing practices. While tests such as inflammatory markers have low predictive value, [23] the fact that they were being carried out suggests GPs may have had a raised index of suspicion for AS in at least some patients.

### Comparison with existing literature

Previous studies of the clinical features of AS have used patient self-report rather than entries coded in GP records [4, 5, 24]. One recent machine learning study used a broadly similar range of single items from a different clinical dataset [12] but did not explicitly code composites which were clinically intuitive. We are not aware of studies which have looked for combinations of features. Our analysis of the emergence of clinical features over time confirms that in some cases there is an observably long time to diagnosis but also shows that the predictive value of clinical features does increase with proximity to diagnosis [12].

### Implications for research and practice

The ultimate purpose of this research is to identify clinically useful predictors of a diagnosis of AS in order to facilitate early diagnosis. None of the features examined here are sufficient on their own, but merit examination in a larger dataset. However some features (such as the multiple episodes of axial pain) may be useful triggers to clinicians to ask more specific questions about inflammatory back pain. Diagnostic support prompts are more effective at the beginning of a consultation (influencing the clinician’s prior probabilities and triggering specific questions) than at the end [25] so computation of the composite indicators of the kind we found, might not need to be carried out in real-time, but could run during quiet periods for the database and be used to inform future consultations.

The next step in research is to repeat this work with a larger, more recent and less sparse dataset. This should be able to access more information about diagnostic tests (through automatic transfer of results from laboratories to electronic records in general practice) and linkage to hospital records (to confirm diagnosis). Additionally, machine learning techniques [12, 26, 27] have potential value in feature reduction and model selection. Ultimately the aim must be to apply these observations within predictive models for earlier referral and diagnosis of AS.

## Conclusion

We have developed and tested conventional and new composite pointers to a diagnosis of ankylosing spondylitis in GP records. Some of these were present several years before the diagnosis and may be valuable targets for systems to support earlier diagnosis.

## Supplementary information

**Additional file 1.**

## Data Availability

The datasets generated during and/or analysed during the current study are not publicly available as they represent anonymised data from clinical records. They are available from the corresponding author on reasonable request and subject to the approval of the Research Applications and Data Management Team at the University of Aberdeen.

## Abbreviations

AS: Ankylosing spondylitis
CI: Confidence interval
ESR: Erythrocyte sedimentation rate
FBC: Full blood count
GP: General practitioner
MRI: Magnetic resonance imaging
NSAID: Non steroidal anti-inflammatory drug
OR: Odds ratio
PCCIU: Primary Care Clinical Informatics Unit
PTI: Practice Team Information.
SSRI: Selective serotonin reuptake inhibitors
