# Supplementary material for: Clinical features in primary care electronic records before diagnosis of ankylosing spondylitis: a nested case-control study
Source: BMC Fam Pract. 2020 May 6;21:78. doi: 10.1186/s12875-020-01149-2 (PMC7201706; doi:10.1186/s12875-020-01149-2)
Supplement: Supplementary file 1 — Additional file 1. [file 12875_2020_1149_MOESM1_ESM.docx]

READ CODES USED

**Symptoms:**

| Where Readcode in:  16A..% Neck pain *  16C.. % Back pain*  N13.. %Other upper spine problems  N14.. % Other lower spine problems  N21.. % Enthesopathy  N11.. % Other Spondylosis  N12.. % Disc Disorder  N094. %Arthralgia  F44.. % Iritis  1A…% Genitourinary symptoms |  |
| --- | --- |

**Procedures:**

| Where Readcode in:  7J3..% Lumbar procedures |  |
| --- | --- |

**Prescriptions:**

| **Has** a prescription for  BNF 10.1.1 NSAIDs  BNF 4.3 Antidepressant drugs  BNF 4.7.1 Non-opioid analgesics  BNF 4.7.2 Opioid analgesics  BNF 10.1.3 DMARDs  BNF 8.2.1 Azathioprine  BNF 6.3.2 Glucocorticoids  BNF 9.1.1 Iron Deficiency Anaemias  BNF 9.1.2 Megalobastic Anaemias |  |
| --- | --- |

**Diagnostic Tests:**

| 424.. % FBC  42B6.% ESR  X7721 CRP  442..% Thyroid hormone tests  44F3.% Alkaline Phos  4KB1.% HLA B27  525.% XR Spine.  527..% XR Pelvis / SIJ  567..% CT Scan  569..% MR Scan |  |
| --- | --- |

**Conditions:**

| N2… % Inflammatory arthritis  N04 % Rheumatoid arthritis  M160. % Psoriatic arthritis  N0450% Juvenile AS  19…% Gastrointestinal symptoms  J4…% Inflammatory Bowel Disease  J521.% IBS  8Cm.. Management of IBS  1682.% Fatigue  R007.% Malaise and fatigue  F286.% CFS  1683.% TATT  E205.%TATT |  |
| --- | --- |
